# Patterns of care, toxicity and outcome in the treatment of salivary gland carcinomas: long-term experience from a tertiary cancer center

**DOI:** 10.1007/s00405-021-06652-5

**Published:** 2021-03-24

**Authors:** Jens von der Grün, Ria Winkelmann, Franz Rödel, Sven Balster, Thomas Neumayer, Shahram Ghanaati, Christian Brandts, Iris Burck, Daniel Martin, Claus Rödel, Nikolina Kesar, Panagiotis Balermpas

**Affiliations:** 1 Department of Radiotherapy and Oncology, University Hospital Frankfurt, Goethe University, Frankfurt, Germany; 2 German Cancer Research Center (DKFZ), Heidelberg, Germany; 3 German Cancer Consortium (DKTK), partner site: Frankfurt/Mainz, Frankfurt, Germany; 4 Senckenberg Institute of Pathology, University Hospital Frankfurt, Goethe University, Frankfurt, Germany; 5 Frankfurt Cancer Institute (FCI), University Hospital Frankfurt, Goethe University, Frankfurt, Germany; 6 Department of Otorhinolaryngology, University Hospital Frankfurt, Goethe University, Frankfurt, Germany; 7 Department of Oral, Maxillofacial and Facial Plastic Surgery, University Hospital Frankfurt, Goethe University, Frankfurt, Germany; 8 Department of Medicine, Hematology/Oncology, University Cancer Center (UCT), University Hospital Frankfurt, Goethe University, Frankfurt, Germany; 9 Department of Diagnostic and Interventional Radiology, University Hospital Frankfurt, Goethe University, Frankfurt, Germany; 10 Department of Radiation Oncology, University Hospital Zurich, Zurich, Switzerland

**Keywords:** Salivary gland carcinoma, Head and neck cancer, Surgery, Radiotherapy, Patterns of care

## Abstract

**Background:**

Salivary gland carcinomas (SGC) cover a heterogeneous group of malignancies with a lack of data of high-level evidence.

**Methods:**

Clinical data of 127 patients treated for SGC at a university cancer center between 2002 and 2017 were analyzed retrospectively. The association of clinicopathological characteristics, treatment modalities, adverse events, and outcome was assessed.

**Results:**

Patients received surgery (*n* = 65), surgery followed by (chemo-)radiotherapy (*n* = 56), or primary (chemo-)radiotherapy (*n* = 6). Injury to the cranial nerves or their branches was the most frequent surgical complication affecting 40 patients (33.1%). Ten year overall and progression-free survival rates were 73.2% and 65.4%, respectively. Parotid tumor site, advanced tumor, and positive nodal stage remained independent negative prognostic factors for overall survival, loco-regional and distant tumor control in multivariate analysis.

**Conclusions:**

Optimizing treatment strategies for SGC, depending on distinct clinicopathological factors, remains challenging due to the low incidence rates of the disease.

**Supplementary Information:**

The online version contains supplementary material available at 10.1007/s00405-021-06652-5.

## Introduction

Salivary gland carcinomas (SGC) account for 1–6% of all head and neck tumors and comprise a variety of 20 different histological subtypes as classified by the World Health Organization (WHO) in 2017 [1, 2]. Some of those subtypes are extremely rare, which prohibits clinical trials being conducted, and thus the development of evidence-based guidelines for treatment standardization [1]. Seventy percent of SGC arise from the parotid gland, while 20–25% derive from the submandibular and sublingual glands. Whereas acinic cell carcinoma (ACC, 15–17%), adenoid cystic carcinoma (AdCC, 16–27%) and mucoepidermoid carcinoma (MEC, 15–38%) represent frequent histopathological entities in the paired, major salivary glands, AdCC and MEC are also common in the minor salivary glands of the oral cavity [3]. While ACC depicts a low-risk malignancy, other histopathological subtypes such as AdCC, salivary duct carcinoma (SDC), or adenocarcinoma—not otherwise specified (ACA) are high-risk tumors with different prognosis [1]. By actual guidelines [4], surgical resection is the standard treatment for SGC including neck dissection (ND) in case of adverse features such as advanced tumor stages (T3–T4) and/or clinically positive lymph nodes of the neck. A common therapeutic challenge, even for high-volume centers, are the positive resection margins often described in the literature, which often lead to the decision for adjuvant/additive treatment [5–7]. Postoperative radiotherapy (PORT) is recommended in case of adverse pathological features (e.g., advanced tumor stages T3–T4, positive resection margins), and AdCC [8]. Various irradiation modalities have been used in the past, including particle-radiotherapy characterized by a high linear energy transfer (LET) and relative biological efficacy (RBE), with heavy ions and neutrons showing some promising results even for radioresistant histopathological entities [9, 10]. Due to the lack of prospective, randomized clinical trials, the benefit of multimodal protocols has not been proven, yet. Data on the efficiency of chemotherapy (CTX), targeted therapy, and immunotherapy for SGC are rare, and so far, without prospective evidence for improved outcome [8, 11–14]. This study aims to analyze patterns of care and the efficacy of surgery, radiotherapy (RT), and chemoradiotherapy (CRT) as single or combined modalities in the treatment of SGC with a focus on the outcome and adverse events from a longstanding experience of a university cancer center.

## Patients and methods

### Patient cohort and tumor staging

Clinical records of 127 patients, treated for primary or recurrent SGC between 2002 and 2017 at the Departments of Otorhinolaryngology, Oral, Maxillofacial and Facial Plastic Surgery, and Radiotherapy and Oncology at the University Hospital Frankfurt, Germany were analyzed retrospectively following institutional ethics board approval (No. 30/17, Ethics Committee, University Hospital Frankfurt, Germany) in accordance with the Declaration of Helsinki. Patients provided written informed consent for scientific use of their pseudonymized data and biomaterial. Routine pretherapeutic staging included physical examination, computed tomography/magnetic resonance imaging (CT/MRI) of the head and neck, biopsy and either CT of the thorax and abdomen, or chest radiography and abdomen sonography prior to treatment. All cases were discussed at an interdisciplinary organized tumor board of the head and neck specialties (otorhinolaryngology, oral and maxillofacial surgery, radiation oncology, medical oncology, pathology, radiology), and all patients provided written informed consent for their individual designated treatment.

Current literature distinguishes between high- and low-risk SGC. Based on this categorization, the tumors of this cohort were also divided into these two groups. The high-risk tumor subgroup comprised ACA, AdCC, SDC, squamous cell carcinoma (SCC), MEC G3, carcinoma ex pleomorphic adenoma, and oncocytic carcinoma. The low-risk tumor subgroup consisted of ACC, MEC G1/G2, polymorphous adenocarcinoma, epithelial–myoepithelial carcinoma, myoepithelial carcinoma, clear cell carcinoma, and basal cell adenocarcinoma [1, 15].

In case of patients with SCC, the final diagnosis was made upon exclusion of any other primary tumor site, including clinical examination, specifically dermatological inspection, and radiological examination. If no squamous cell primary was detectable after thorough staging, the cases were included in the present analysis, as the treatment was identical to primary parotideal malignancies.

### Treatment protocols

Surgical procedures included tumor resection, whereas ND was conducted for advanced tumor stages (T3–T4), and/or clinically positive lymph nodes of the neck. As mentioned before by Quer et al. [16], total parotidectomy in this study was defined as parotidectomy with preservation of the facial nerve, whereas radical parotidectomy included parotidectomy with sacrifice of the facial nerve. As further reported by previous studies, in some cases, the probability of occult cervical lymph node metastases might be given in up to 31.2% [17]. Thus, the option of elective ND in T1N0- and T2N0-staged patients was critically discussed with each of the respective individuals.

RT was delivered either as three-dimensional conformal radiotherapy (3D-RT), or, since 2010, as intensity-modulated radiotherapy (IMRT), both under image guidance, implementing portal imaging and/or conebeam CT scans. Each patient received a planning CT and thermoplastic masks were used for immobilization during irradiation (photon energy: 6 MV). The planned target volumes (PTV) included elective irradiation of the draining cervical lymph nodes up to 50–54 Gy, dose escalation up to 58–60 Gy for involved lymph node levels, and a boost to the primary tumor region with a median total reference dose of 64–66 Gy for PORT and postoperative chemoradiotherapy (POCRT), and 70–72 Gy for definitive treatment. PORT or POCRT was applied to patients with adverse pathological features, such as G3 histology, advanced tumor stages (T3–T4), positive lymph nodes, positive resection margins, neural/perineural invasion, and lymphatic/vascular invasion.

Concomitant CRT was only applied in case of loco-regionally advanced ACA or SCC with additional risk factors such as close or positive resection margins and extra-nodal extension. Acute toxicities were evaluated according to the Common Terminology Criteria for Adverse Events (CTCAE) of the National Cancer Institute, Bethesda, MD, USA in their, respectively, current version by the time of treatment. Due to the retrospective character of the analysis and scarce documentation of late toxicities, the latter were not considered further throughout the study. Follow-up appointments, including clinical examination, CT/MRI scans of the head and neck, as well as additional biopsy in case of suspicious findings were scheduled every 3 months for the first 2 years, and every 6 months thereafter for a total of 5 years.

### Statistical analysis

Statistical analyses were performed using SPSS (IBM SPSS Statistics, v24.0, Armonk, NY, USA) and R (The R Foundation for Statistical Computing, v3.5.0, Vienna, Austria). The main oncological outcome measures were overall survival (OS), loco-regional progression-free survival (LPFS), distant metastases-free survival (DMFS), and progression-free survival (PFS). The endpoints since the date of diagnosis were set as the occurrence of the respective event or death from any cause, while patients, who were alive and/or event-free, were censored at the last contact. The log-rank test was used with respect to univariate analyses, whereas the Cox model was applied considering multivariate analyses. Variables that were evaluated significant in the univariate model were forwarded to the multivariate one in a single step, following the Schoenfeld test for proportional hazards. To avoid overfitting [18], we aimed for a minimum of ten events per variable in multivariate analysis. Accordingly, age as a multi-influenced variable and any non-categorial variable with an obvious influence on survival endpoints were excluded a priori from the Cox model. Additionally, differences between categorial variables were assessed by the Pearson chi-squared test. Survival curves were visualized by the Kaplan–Meier method. Statistical significance was considered at *p* ≤ 0.05.

## Results

### Clinicopathological characteristics and outcome

Sixty-nine out of 127 patients (54.3%) were male, and the overall median age was 61 years at the time of diagnosis. The majority of the SGC were located in the parotid gland (72.4%), followed by 18.1% in the minor salivary glands. Details on initial tumor stages, resection margins, treatment modalities, and histopathological entities are shown in Table 1.

**Table 1:** Clinicopathological characteristics

Clinicopathological characteristics	*n* (%)
Total	127 (100.0)
Gender	
Male	69 (54.3)
Female	58 (45.7)
Age^a^	
< Median	62 (48.8)
≥ Median	65 (51.2)
Tumor site	
Parotid gland	92 (72.4)
Submandibular gland	10 (7.9)
Sublingual gland	2 (1.6)
Minor salivary glands	23 (18.1)
T-stage	
T1	44 (37.6)
T2	17 (14.5)
T3	31 (26.5)
T4	25 (21.4)
Missing values	10
N-stage	
N0	84 (66.1)
N1	18 (14.2)
N2	24 (18.9)
N3	1 (0.8)
M-stage	
M0	108 (85.0)
M1	4 (3.1)
MX^b^	15 (11.8)
Resection margins	
R0	77 (63.6)
R1	37 (30.6)
R2	7 (5.8)
No surgery	6
Treatment modalities	
Surgery alone	62 (48.8)
Neoadjuvant chemotherapy^c^ and surgery	3 (2.4)
Neoadjuvant chemotherapy^d^ and surgery followed by radiotherapy	2 (1.6)
Surgery followed by radiotherapy	41 (32.3)
Surgery followed by chemoradiotherapy	13 (10.2)
Primary radiotherapy	5 (3.9)
Primary chemoradiotherapy	1 (0.8)
Carcinoma histology	
Acinic cell	12 (9.4)
Mucoepidermoid G1/G2	20 (15.7)
Mucoepidermoid G3	2 (1.6)
Adenoid cystic	24 (18.9)
Polymorphous adeno	2 (1.6)
Epithelial–myoepithelial	4 (3.1)
Clear cell	1 (0.8)
Basal cell adeno	4 (3.1)
Adeno, not otherwise specified	31 (24.4)
Salivary duct	7 (5.5)
Myoepithelial	2 (1.6)
Ex pleomorphic adenoma	2 (1.6)
Squamous cell	15 (11.8)
Oncocytic	1 (0.8)

^a^Age: median 61 years, range 9–93 years

^b^MX was defined as any suspect radiographic finding requiring process control

^c^TPF (docetaxel, cisplatin, 5-fluorouracil), *n* = 2; missing information on sequence and agent, *n* = 1

^d^TPF (docetaxel, cisplatin, 5-fluorouracil), *n* = 2

**Fig. 1:**
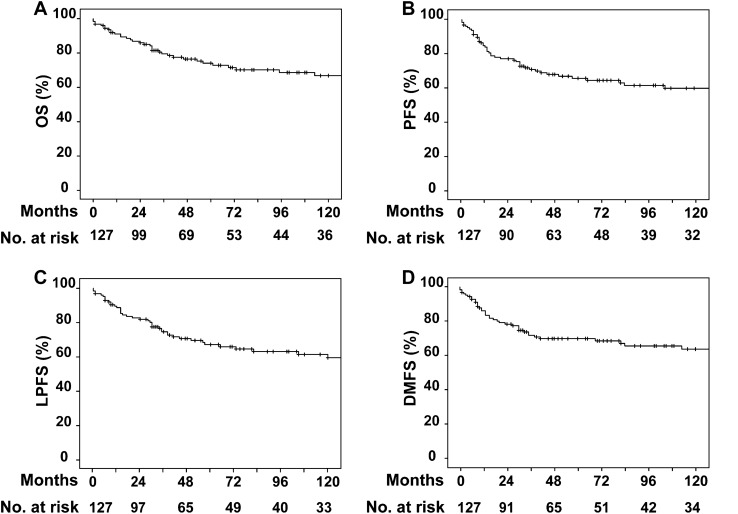
Oncological outcome of total cohort. Abbreviations: *OS* overall survival, *PFS* progression-free survival, *LPFS *loco-regional progression-free survival, *DMFS* distant metastases-free survival

The median overall follow-up period was 55 months (range: 0–443 months) and reached 74 months (range: 1–443 months) in patients who were alive by the time of their last visit. Until the end of follow-up, 17.3% of the patients alive developed loco-regional recurrences and 17.3% of the surviving patients were diagnosed with distant metastases (Fig. 1). Median OS since the date of primary diagnosed loco-regional recurrence or distant metastases was 28.5 months (range: 0–233 months) and 21.5 months (range: 1–130 months), respectively.

### Treatment procedures and adverse events

A total of 121 patients (95.3%) received surgery of whom 84 individuals (69.4%) were treated by parotidectomy. Sixty-eight patients (56.2%) received at least ipsilateral ND, whereas bilateral ND was performed predominantly in patients with midline crossing tumors.

Injury to the cranial nerves (V, VII, X, XI, and XII) or their branches of any severity grade, as classified by Seddon and Sunderland [19, 20], was the most frequent surgical complication affecting a total of 40 patients (33.1%) at follow-up. Particularly, iatrogenic facial nerve damage or sacrifice within the scope of radical parotidectomy represented the most common (*n* = 32, 80.0%) of all neurological complications. Reconstructive surgery and symptomatic treatment of facial nerve palsy included local and/or distant nerve graft transfer (*n* = 3), primary nerve coadaptation (*n* = 2), upper eyelid gold weight placement (*n* = 3), tarsorrhaphy (*n* = 2), and/or lateral tarsal strip procedure (*n *= 1). Due to the retrospective character of our study, a detailed re-assessment on the extent of facial nerve involvement and hence a rating according to the scale by House and Brackmann was not possible [21]. Wound healing disorders occurred in six patients (5.0%), including wound dehiscence, fistula, and Frey’s syndrome (*n* = 2 each), while postoperative hemorrhage was observed in three patients (2.5%) (Table 2). However, the addition of ND to surgery was well tolerated and did not increase the surgical complication rate compared to tumor resection alone (*p* = 0.069).

**Table 2:** Applied surgical techniques and coinciding peri- and postoperative complications

Surgical techniques	*n* (%)
Treated patients	121 (100.0)
Main procedure	
Lateral (≙ superficial) parotidectomy	14 (11.6)
Total parotidectomy	36 (29.8)
Radical parotidectomy	12 (9.9)
Parotidectomy, not specified	22 (18.2)
Submandibular sialadenectomy	11 (9.1)
Sublingual sialadenectomy	1 (0.8)
Local resection	16 (13.2)
Resection including partial maxillectomy	6 (5.0)
Resection including partial mandibulectomy	3 (2.5)
Management of the neck	
Ipsilateral neck dissection	68 (56.2)
Bilateral neck dissection	12 (9.9)
Neck dissection, not specified	12 (9.9)
None	29 (24.0)

Surgical complications	*n* (%)	*n* (%)
Treated patients	121 (100.0)	
Injury to the cranial nerves or their branches		
Yes, including	40 (33.1)	40 (100.0)
Lingual nerve (V)		2 (5.0)
Facial nerve (VII)		32 (80.0)
Recurrent laryngeal nerve (X)		1 (2.5)
Accessory nerve (XI)		1 (2.5)
Hypoglossal nerve (XII)		4 (10.0)
No	81 (66.9)	
Postoperative hemorrhage		
Yes	3 (2.5)	
No	118 (97.5)	
Wound-healing disorder		
Yes	6 (5.0)	
No	115 (95.0)	

Overall RT completion rate reached 98.4%. A single patient refused to continue PORT at 11.94 Gy of the prescribed 64.02 Gy without experiencing any adverse events. Irradiation was applied by photon therapy for the vast majority of patients (*n* = 53), the median applied dose to the primary tumor region was 64.8 Gy regarding PORT and 64.0 Gy regarding POCRT.

CTX regimens in case of the 13 patients receiving POCRT comprised cisplatin and 5-fluorouracil (5-FU) (*n* = 7), carboplatin and 5-FU (*n* = 1), cisplatin monotherapy (*n* = 1), 5-FU monotherapy (*n* = 3) and one unknown CTX regimen as treated prior to referral. There was no difference regarding oncological outcome comparing PORT with POCRT (OS: *p* = 0.236, LPFS: *p* = 0.609, DMFS: *p* = 0.277; data not shown). A single patient treated by primary CRT received cisplatin and 5-FU. The only common grade ≥ II toxicities observed after RT was dermatitis and mucositis in the majority of the irradiated patients, amounting to 8.1% (grade ≥ III 1.6%) and 19.4% (grade ≥ III 1.6%), respectively, whereas leukocytopenia grade ≥ III in 15.4% was the most relevant adverse event of the patients treated with concomitant CTX. Neither grade IV adverse events nor patient deaths related to RT/CRT occurred (Supplementary Table 1).

### Association of clinicopathological characteristics and treatment modalities

**Fig. 2:**
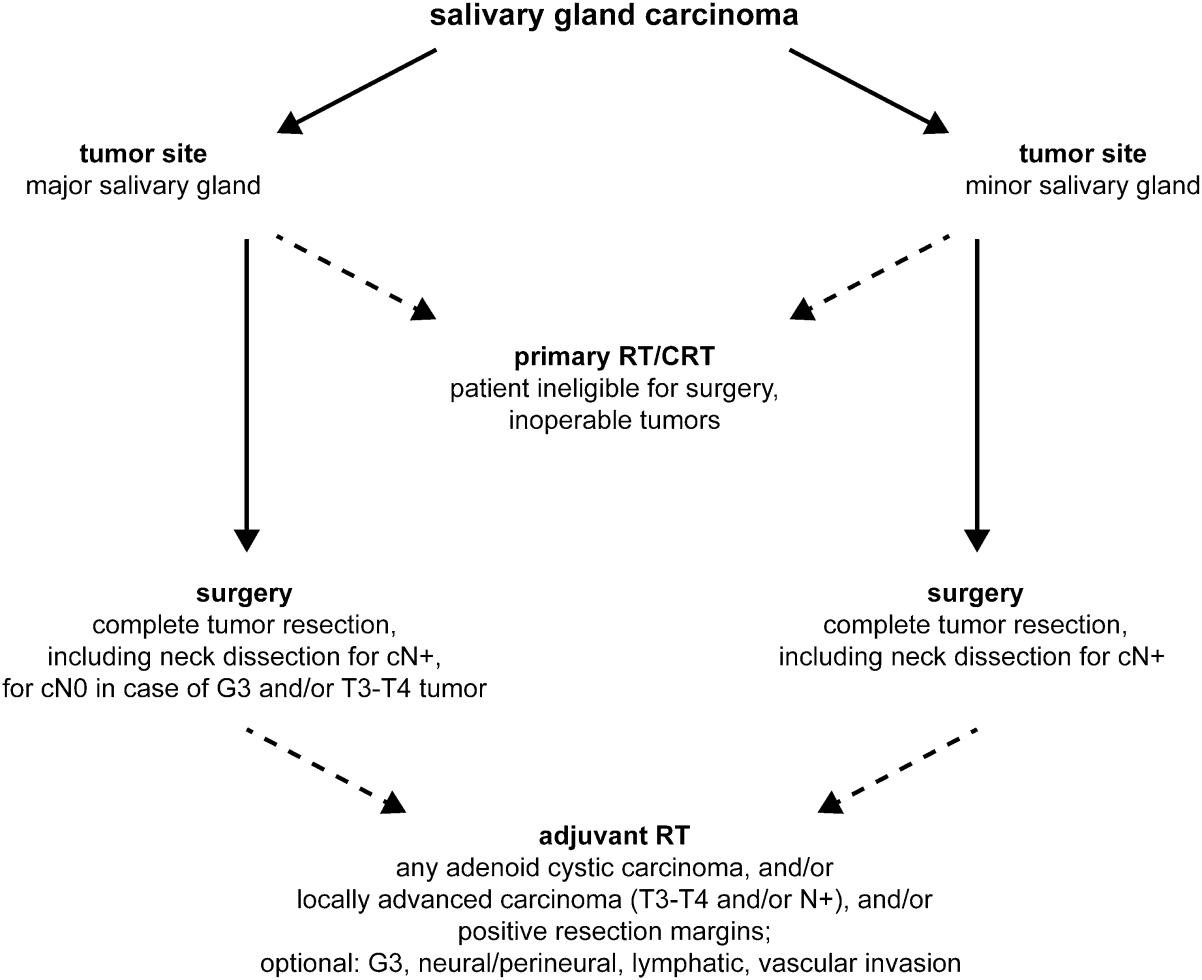
Treatment of salivary gland carcinomas. Abbreviations: *RT* radiotherapy, *CRT* chemoradiotherapy

The association of clinicopathological characteristics and treatment modalities is shown in Supplementary Table 2. Based on the treatment patterns and the most common procedures applied in our patient cohort, we reconstructed a fluxogram (Fig. 2), which was found to be in accordance with the most recent version of the National Comprehensive Cancer Network (NCCN) guidelines for these malignancies [4].

Tumors of the parotid and submandibular glands were treated more often by surgery to both the primary and the neck, than tumors arising from the sublingual gland and minor salivary glands (*p* = 0.018). Nevertheless, additional treatment by PORT/POCRT was not influenced by the tumor site (*p* = 0.566). Patients with loco-regionally advanced (*p* = 0.011) and high-risk tumors (*p* < 0.001), nodal involvement (*p* = 0.009), and positive resection margins (*p* = 0.004) received surgery and PORT/POCRT more often (Supplementary Table 2).

### Impact of tumor site and histopathology on initial tumor stage

Tumors of the parotid gland were more common in older patients (*p* = 0.006). Furthermore, their resection was more often associated with ND (*p* = 0.008). Also, the histopathological subtypes were heterogeneously distributed between the different salivary glands (*p* = 0.001) (Supplementary Table 3).

Regarding the five most frequently occurring histopathological subtypes within the cohort, the diagnosis of ACA and SCC was associated with higher age (*p* < 0.001), as well as with advanced tumor (*p* < 0.001), and nodal stage (*p* < 0.001 (Supplementary Table 4).

### Impact of clinicopathological characteristics on outcome

In univariate analysis, higher age, primary tumor location within the parotid gland, tumor histopathology, advanced tumor and positive nodal stage, as well as positive resection margins were associated with impaired oncological outcome in terms of OS, LPFS, DMFS and PFS. Furthermore, adjuvant RT was associated with poor DMFS and PFS. Tumor site (parotid gland), advanced tumor and positive nodal stage remained independent prognostic factors in multivariate analysis regarding reduced OS, PFS, LPFS, and DMFS (Table 3).

**Table 3:** Prognostic factors for overall, loco-regional progression-free, distant metastases-free and progression-free survival

Clinicopathological characteristics	Univariate analysis	Schoenfeld test	Multivariate analysis		
	*p*	*p*	Hazard ratio	95% Confidence interval	*p*
Overall survival (*n* = 36 events included into multivariate analysis)		0.108 (global)			
Gender (male vs. female)	0.188				
Age^a^ (< median vs. ≥ median)	**<0.001**				
Tumor site (parotid gland vs. other)	**0.007**	0.389	0.301	0.110–0.825	**0.020**
Histology (low-risk vs. high-risk)	**0.001**	**0.008**			
T-stage (T1–T2 vs. T3–T4)	**<0.001**	0.536	5.476	2.297–13.054	**<0.001**
N-stage (N0 vs. N1–N3)	**<0.001**	0.659	3.067	1.462–6.431	**0.003**
Resection margins (R0 vs. other)	**0.001**	0.229	1.574	0.792–3.132	0.196
Surgery (with neck dissection vs. without neck dissection)	0.603				
Adjuvant radiotherapy (yes vs. no)	0.141				
Loco-regional progression-free survival (*n* = 44 events included into multivariate analysis)		0.684 (global)			
Gender (male vs. female)	0.194				
Age^a^ (< median vs. ≥ median)	**<0.001**				
Tumor site (parotid gland vs. other)	**0.001**	0.992	0.180	0.065–0.501	**0.001**
Histology (low-risk vs. high-risk)	**0.005**	0.607	1.038	0.397–2.712	0.940
T-stage (T1–T2 vs. T3–T4)	**<0.001**	0.646	5.206	2.383–11.373	**<0.001**
N-stage (N0 vs. N1–N3)	**<0.001**	0.637	2.528	1.194–5.352	**0.015**
Resection margins (R0 vs. other)	**0.009**	0.094	1.121	0.588–2.134	0.729
Surgery (with neck dissection vs. without neck dissection)	0.431				
Adjuvant radiotherapy (yes vs. no)	0.097				
Distant metastases-free survival (*n* = 40 events included into multivariate analysis)		0.190 (global)			
Gender (male vs. female)	0.096				
Age^a^ (< median vs. ≥ median)	**<0.001**				
Tumor site (parotid gland vs. other)	**0.006**	0.870	0.313	0.116–0.846	**0.022**
Histology (low-risk vs. high-risk)	**<0.001**	**0.018**			
T-stage (T1–T2 vs. T3–T4)	**<0.001**	0.698	5.053	2.201–11.601	**<0.001**
N-stage (N0 vs. N1–N3)	**<0.001**	0.446	3.718	1.773–7.794	**0.001**
Resection margins (R0 vs. other)	**0.001**	0.102	1.572	0.814–3.034	0.178
Surgery (with neck dissection vs. without neck dissection)	0.456				
Adjuvant radiotherapy (yes vs. no)	**0.024**	0.414	1.247	0.616–2.524	0.540
Progression-free survival (*n* = 46 events included into multivariate analysis)		0.741 (global)			
Gender (male vs. female)	0.108				
Age^a^ (< median vs. ≥ median)	**<0.001**				
Tumor site (parotid gland vs. other)	**0.001**	0.956	0.193	0.071–0.529	**0.001**
Histology (low-risk vs. high-risk)	**0.002**	0.764	1.092	0.421–2.831	0.857
T-stage (T1–T2 vs. T3–T4)	**<0.001**	0.497	4.583	2.158–9.733	**<0.001**
N-stage (N0 vs. N1–N3)	**<0.001**	0.706	2.857	1.348–6.055	**0.006**
Resection margins (R0 vs. other)	**0.007**	0.110	1.182	0.629–2.223	0.603
Surgery (with neck dissection vs. without neck dissection)	0.359				
Adjuvant radiotherapy (yes vs. no)	**0.044**	0.380	1.125	0.587–2.160	0.722

^a^Age: median 61 years, range 9–93 years; factor was excluded a priori from multivariate analysis as a multi-influenced variable

## Discussion

To date, only a few large retrospective studies regarding the treatment of SGC have been published and to the best of our knowledge, there are no prospective trials reporting results on primary treatment modalities including surgery or radiotherapy. Besides, most of the retrospective trials either report on major or minor salivary glands or distinct histopathological subtypes, exclusively [5, 22–25].

The current study provides comprehensive data on the long-term outcome with a median follow-up period of 55 and 74 months for those patients who were alive by the time of their last appointment. We consider this to be a major strength of the study, as long-term follow-up has been recommended to detect local recurrences or metastases occurring beyond 60 months after initial treatment [26]. Furthermore, to prevent selection bias, all treated patients presented in the cancer center during the period analyzed were included in analysis.

The cohort of patients enrolled is largely heterogeneous, as expected for this diagnosis, however, representative of the daily clinical practice in a large university cancer center. The median age by the time of initial tumor diagnosis and the slight predominance of the male gender is in accordance with two large retrospective cohorts encompassing different entities of SGC, reporting overall median ages of 58 and 63 years and proportions of male patients to be 52.0% and 58.5%, respectively [6, 7]. Similar to one of the largest studies on SGC by Terhaard et al. on behalf of the Dutch Head and Neck Oncology Cooperative Group retrospectively including 565 patients [7], and a follow-up study by Westergaard-Nielsen et al. including 1601 SGC patients [27], the tumors in our cohort were more likely to be diagnosed at T1/T2-stage and N0-stage without signs of distant metastases. Furthermore, in this cohort, AdCC, ACA and MEC were the predominant histological subtypes, in accordance with the literature [7]. Interestingly, the percentage of SCC in our cohort is 11.8% and higher compared to previous reports (4%) [1, 28], so that false clinical diagnosis as an SGC primary instead of metastasis from other tumors at least for a part of these cases cannot be ruled out. Primary SCC of the salivary glands, mostly arising from Stensen’s and Wharton’s ducts is mostly caused by chronic inflammation as result of e.g., chronic sialolithiasis and has been described before [1].

Complete tumor resection in our study was achieved in 63.6% of the cases, slightly more often than reported by Terhaard et al. where complete primary tumor resection was achieved in 59% of the cases [7]. Incomplete primary tumor resection is not uncommon and complete resection rates as low as 31.3% have been reported even for experienced and high-volume centers [5, 6]. Parotid tumors in this study were more often diagnosed at advanced patient age (*p* = 0.006). ACA and SCC occurred more often in the parotid glands and their diagnosis has been associated with higher age before [7].

Ten year OS rate was 73.2% in our cohort, which is higher as reported in literature ranging from 43 to 52% [5–7, 25, 27, 29, 30]. Independent prognostic factors for OS in the present study were parotid tumor site, advanced T- and positive N-stage. Yet, tumor site has been associated with OS in multivariate analysis by other authors as well [7, 31]. Moreover, similar to our data, Bjørndal et al. stressed the prognostic importance of tumor stage for recurrence and survival [30]. Possible explanations for the higher OS rate in our cohort might be the inclusion of predominantly high-risk subtypes by other authors [25], or older patient series ranging back to the 1940s with a lack of modern diagnostic imaging using different RT techniques [5–7, 29].

Primary RT/CRT was performed when patients were ineligible for primary surgery. A review by Wang et al. revealed significant impairment of OS for primary RT when compared to PORT in five series with a total of 913 patients [8]. Due to patients’ risk profiles, ND was more often performed for parotid gland tumors than for other tumor locations (*p* = 0.008). Performing ND did not have a significant impact on OS. POCRT in this series was applied in cases of loco-regionally advanced ACA or SCC with additional risk factors like close or positive resection margins and extra-nodal extension and concomitant CTX was platin-based in combination with 5-FU in most cases. Despite the unfavorable tumor characteristics outcome was not impaired for these patients when compared to the group receiving only PORT. Indeed, from the cytotoxic regimens tested with or without RT for SGC, cisplatin and 5-FU were shown to be amongst the most efficient agents within various small patient series for locally advanced or recurrent/metastatic disease [8, 11]. While some authors reported good local control rates for POCRT, Hsieh et al. reported improved local control rates for POCRT (*n* = 58) in comparison to PORT (*n* = 33) [32–34]. However, a SEER—Surveillance, Epidemiology, and End Results—Medicare database analysis revealed increased mortality and toxicity for the concomitant POCRT when compared to PORT alone [35]. Due to heterogeneous patient cohorts and individual indications for POCRT, interpretation of such data remains difficult. Taken together, no evidence exists for an additional benefit of CTX combined with RT in the postoperative setting. The main hematological toxicities are non-negligible and, therefore, the prescription of such regimens should be decided critically and reserved for selected, individual cases.

LPFS rate after 10 years was 66.9% in our cohort, which is in line with the literature. In analyses encompassing different histological subgroups and primary tumor sites, likewise this study, LPFS varied between 59 and 88% at 10 years [5–7, 25, 27, 36]. Various series reported improved loco-regional control for the combination of surgery and PORT [7, 37, 38]. However, in our cohort, the outcome was not affected by the implementation of PORT in multivariate analysis, although PORT was associated with both improved DMFS and PFS in univariate analysis. In accordance with our multivariate analysis, primary tumor site (parotid gland), advanced T- and positive N-stage have been reported to impair LPFS [6, 7, 31].

In the literature, the most common failures are distant metastases. Reported rates range from 15 to 37% after 10 years which is in line with our results [5–7, 29, 36, 37, 39, 40]. Primary tumor site, loco-regionally advanced tumor, and nodal stage were associated with DMFS in multivariate analysis, as reported before [6, 7, 36, 40–42].

In our cohort, adverse events following surgical intervention often comprised injuries of the cranial nerves or their branches (33.1%) with facial nerve injuries being the most common (80.0%), whereas postoperative hemorrhage and wound-healing disorders were rare. Several authors reported on rates of facial nerve injuries from parotidectomies and numbers range between 29.2% and 45.0%, yet [29, 43–45]. CRT/RT was well tolerated with low grade ≥ III toxicity rates compared to the literature (7.4–16.0%), which might be related to the broader implementation of IMRT and image-guidance in our cohort [5, 6, 24]. Dermatitis and mucositis of any grade were the most common RT-related sequelae and occurred as expected in almost any patient. No unexpected or grade IV–V acute toxicities occurred in this cohort and all patients but one completed the prescribed course, which may indicate that modern techniques can improve tolerability of this treatment modality [23, 29].

This study has several limitations: first, its retrospective character. Second, the heterogeneity of the patient cohort in terms of tumor site, histopathological entities and treatment hamper comparability and the derivation of treatment recommendations. Third, low patient numbers for rare tumor entities. Fourth, the pathological parameters vascular and lymphatic invasion, perineural invasion as well as extra-nodal extension were not analyzed. Fifth, possible referral bias. Sixth, the inclusion of some very advanced/palliative cases, which might have distorted the results. Although there are limitations to this study as mentioned before, this is one of the most recent series regarding the treatment of SGC from a tertiary tumor center, implementing all current techniques of surgery and radiotherapy with a comparably long follow-up period.

## Conclusions

SGC are a heterogeneous group of carcinomas with varying outcome depending on factors such as primary tumor site and tumor stage. Optimizing multimodal treatment strategies for distinct histological entities, tumor localizations, and stages remains challenging due to low incidence rates. Radical surgery remains the mainstay of treatment, followed by radiotherapy in case of adverse pathological features, and results in fairly good survival rates, at least in high-volume centers. The role of chemotherapy remains controversial. However, to further improve outcome and reduce toxicities, prospective clinical trials are warranted. Large, international, prospective registers and systematic retrospective meta-analyses are necessary for overcoming recruiting problems for the rarest entities.

## Supplementary Information

Below is the link to the electronic supplementary material.

Supplementary file1 (DOCX 49 KB)

## Data Availability

The datasets generated and/or analyzed during the current study are available from the corresponding author on reasonable request.
