# Supplementary material for: Patterns of care, toxicity and outcome in the treatment of salivary gland carcinomas: long-term experience from a tertiary cancer center
Source: Eur Arch Otorhinolaryngol. 2021 Mar 24;278(11):4411–21. doi: 10.1007/s00405-021-06652-5 (PMC8486723; doi:10.1007/s00405-021-06652-5)
Supplement: Supplementary file 1 — Supplementary file1 (DOCX 49 KB) [file 405_2021_6652_MOESM1_ESM.docx]

**Supplementary table 1.** Dose prescriptions and adverse events during primary and adjuvant (chemo-)radiotherapy.

| Dose prescriptions of radiotherapy | Postoperative radiotherapy | Postoperative chemoradiotherapy | Primary radiotherapy | Primary chemoradiotherapy | |
| --- | --- | --- | --- | --- | --- |
| Total n subgroup  (% of total cohort) | 43 (33.9) | 13 (10.2) | 5 (3.9) | 1 (0.8) | |
| Radiotherapy not completed, n (% of subgroup) | 1 (2.3) | 0 (0) | 0 (0) | 0 (0) | |
| Photon therapy (n=57), Gy median (range) | n=40 | n=13 | n=3 | n=1 | |
| Primary tumor region | 64.8 (45.0-70.4) | 64.0 (59.4-69.2) | 70.0 (55.0-70.2) | 72.0 | |
| Involved neck | 59.4 (50.0-60.0) | 59.4 (59.4-59.4) | 58.0 (58.0-58.0) | 59.4 | |
| Elective neck | 50.4 (48.6-54.1) | 50.4 (50.0-54.1) | 50.0 (50.0-50.0) | 50.4 | |
| C12-heavy ion therapy (n=1), Gy median | n=1 |  |  |  | |
| Primary tumor region | 36.0 |  |  |  | |
| Bimodal therapy (n=4), Gy median (range) | n=2 |  | n=2 |  | |
| Primary tumor region, C12-heavy ion boost dose | 21.0 (18.0-24.0) |  | 21.0 (18.0-24.0) |  | |
| Involved neck, photon dose | 55.6 (50.0-61.2) |  | 47.0 (40.0-54.0) |  | |
| Adverse events^a^ | **Primary / postoperative (chemo-)radiotherapy** | | | |  |
| Dermatitis, n (% of total subgroup) |  | | | |  |
| Grade ≤2 | 61 (98.4) | | | |  |
| Grade ≥3 | 1 (1.6) | | | |  |
| Mucositis, n (% of total subgroup) |  | | | |  |
| Grade ≤2 | 61 (98.4) | | | |  |
| Grade ≥3 | 1 (1.6) | | | |  |
| Chemotherapy related toxicities,  n (% of chemoradiotherapy subgroup) |  | | | |  |
| Anemia grade ≥3 | 0 (0) | | | |  |
| Leukocytopenia grade ≥3 | 2 (15.4) | | | |  |
| Thrombocytopenia grade ≥3 | 0 (0) | | | |  |

^a^ Grading according to the National Cancer Institute (NCI) Common Terminology Criteria for Adverse Events (CTCAE) current version by the time of treatment.

**Supplementary table 2.** Primary treatment modalities.

| Clinicopathological characteristics | Surgery | |  | Surgery | |  | Postoperative radiotherapy | |  | | Primary radiotherapy | | |
| --- | --- | --- | --- | --- | --- | --- | --- | --- | --- | --- | --- | --- | --- |
|  | **Without neck dissection** | **With**  **Neck dissection** | **p^a^** | **Without postoperative**  **(chemo)-radiotherapy** | **With postoperative (chemo)-radiotherapy** | **p^a^** | **Postoperative radiotherapy** | **Postoperative chemoradiotherapy** | **p^a^** | **Radiotherapy^b^** | | **Chemoradiotherapy^b^** |  |
| Total n = 127 (%) | 29 (24.0) | 92 (76.0) |  | 65 (53.7) | 56 (46.3) |  | 43 (76.8) | 13 (23.2) |  | 5 (83.3) | | 1 (16.7) |  |
| Gender |  |  |  |  |  |  |  |  |  |  | |  |  |
| Male | 13 (20.0) | 52 (80.0) |  | 31 (47.7) | 34 (52.3) |  | 24 (70.6) | 10 (29.4) |  | 3 (75.0) | | 1 (25.0) |  |
| Female | 16 (28.6) | 40 (71.4) | 0.271 | 34 (60.7) | 22 (39.3) | 0.152 | 19 (86.4) | 3 (13.6) | 0.172 | 2 (100.0) | | 0 (0) |  |
| Age^c^ |  |  |  |  |  |  |  |  |  |  | |  |  |
| < Median | 16 (25.8) | 46 (74.2) |  | 35 (56.5) | 27 (43.5) |  | 23 (85.2) | 4 (14.8) |  | 0 (0) | | 0 (0) |  |
| ≥ Median | 13 (22.0) | 46 (78.0) | 0.627 | 30 (50.8) | 29 (49.2) | 0.537 | 20 (69.0) | 9 (31.0) | 0.151 | 5 (83.3) | | 1 (16.7) |  |
| Tumor site |  |  |  |  |  |  |  |  |  |  | |  |  |
| Parotid gland | 15 (17.4) | 71 (82.6) |  | 43 (50.0) | 43 (50.0) |  | 32 (74.4) | 11 (25.6) |  | 5 (83.3) | | 1 (16.7) |  |
| Submandibular gland | 2 (20.0) | 8 (80.0) |  | 7 (70.0) | 3 (30.0) |  | 2 (66.7) | 1 (33.3) |  | 0 (0) | | 0 (0) |  |
| Sublingual gland | 1 (50.0) | 1 (50.0) |  | 1 (50.0) | 1 (50.0) |  | 1 (100.0) | 0 (0) |  | 0 (0) | | 0 (0) |  |
| Minor salivary glands | 11 (47.8) | 12 (52.2) | **0.018** | 14 (60.9) | 9 (39.1) | 0.566 | 8 (88.9) | 1 (11.1) | 0.717 | 0 (0) | | 0 (0) |  |
| T-stage |  |  |  |  |  |  |  |  |  |  | |  |  |
| T1 | 14 (32.6) | 29 (67.4) |  | 31 (72.1) | 12 (27.9) |  | 11 (91.7) | 1 (8.3) |  | 1 (100.0) | | 0 (0) |  |
| T2 | 2 (11.8) | 15 (88.2) |  | 7 (41.2) | 10 (58.8) |  | 7 (70.0) | 3 (30.0) |  | 0 (0) | | 0 (0) |  |
| T3 | 3 (10.3) | 26 (89.7) |  | 11 (37.9) | 18 (62.1) |  | 14 (77.8) | 4 (22.2) |  | 2 (100.0) | | 0 (0) |  |
| T4 | 4 (18.2) | 18 (81.8) | 0.090 | 9 (40.9) | 13 (59.1) | **0.011** | 8 (61.5) | 5 (38.5) | 0.351 | 2 (66.7) | | 1 (33.3) |  |
| Missing values | 6 | 4 |  | 7 | 3 |  | 3 | 0 |  | 0 | | 0 |  |
| N-stage |  |  |  |  |  |  |  |  |  |  | |  |  |
| N0 | 29 (36.3) | 51 (63.7) |  | 51 (63.7) | 29 (36.3) |  | 26 (89.7) | 3 (10.3) |  | 3 (100.0) | | 0 (0) |  |
| N1 | 0 (0) | 17 (100.0) |  | 5 (29.4) | 12 (70.6) |  | 8 (66.7) | 4 (33.3) |  | 2 (100.0) | | 0 (0) |  |
| N2 | 0 (0) | 23 (100.0) |  | 8 (34.8) | 15 (65.2) |  | 9 (60.0) | 6 (40.0) |  | 0 (0) | | 1 (100.0) |  |
| N3 | 0 (0) | 1 (100.0) | **<0.001** | 1 (100.0) | 0 (0) | **0.009** | 0 (0) | 0 (0) | 0.056 | 0 (0) | | 0 (0) |  |
| Resection margins |  |  |  |  |  |  |  |  |  |  | |  |  |
| R0 | 19 (24.7) | 58 (75.3) |  | 50 (64.9) | 27 (35.1) |  | 21 (77.8) | 6 (22.2) |  | NA | | NA |  |
| R1 | 7 (18.9) | 30 (81.1) |  | 13 (35.1) | 24 (64.9) |  | 19 (79.2) | 5 (20.8) |  | NA | | NA |  |
| R2 | 3 (42.9) | 4 (57.1) | 0.385 | 2 (28.6) | 5 (71.4) | **0.004** | 3 (60.0) | 2 (40.0) | 0.644 | NA | | NA |  |
| No surgery | NA | NA |  | NA | NA |  | NA | NA |  | 5 (83.3) | | 1 (16.7) |  |
| Carcinoma histology |  |  |  |  |  |  |  |  |  |  | |  |  |
| Acinic cell | 4 (36.4) | 7 (63.6) |  | 7 (63.6) | 4 (36.4) |  | 4 (100.0) | 0 (0) |  | 1 (100.0) | | 0 (0) |  |
| Mucoepidermoid G1/G2 | 6 (30.0) | 14 (70.0) |  | 17 (85.0) | 3 (15.0) |  | 2 (66.7) | 1 (33.3) |  | 0 (0) | | 0 (0) |  |
| Mucoepidermoid G3 | 2 (100.0) | 0 (0) |  | 2 (100.0) | 0 (0) |  | 0 (0) | 0 (0) |  | 0 (0) | | 0 (0) |  |
| Adenoid cystic | 4 (16.7) | 20 (83.3) |  | 9 (37.5) | 15 (62.5) |  | 15 (100.0) | 0 (0) |  | 0 (0) | | 0 (0) |  |
| Polymorphous adeno | 2 (100.0) | 0 (0) |  | 2 (100.0) | 0 (0) |  | 0 (0) | 0 (0) |  | 0 (0) | | 0 (0) |  |
| Epithelial-myoepithelial | 2 (50.0) | 2 (50.0) |  | 4 (100.0) | 0 (0) |  | 0 (0) | 0 (0) |  | 0 (0) | | 0 (0) |  |
| Clear cell | 0 (0) | 1 (100.0) |  | 0 (0) | 1 (100.0) |  | 1 (100.0) | 0 (0) |  | 0 (0) | | 0 (0) |  |
| Basal cell adeno | 2 (50.0) | 2 (50.0) |  | 3 (75.0) | 1 (25.0) |  | 1 (100.0) | 0 (0) |  | 0 (0) | | 0 (0) |  |
| Adeno, not otherwise specified | 5 (17.9) | 23 (82.1) |  | 12 (42.9) | 16 (57.1) |  | 8 (50.0) | 8 (50.0) |  | 2 (66.7) | | 1 (33.3) |  |
| Salivary duct | 0 (0) | 7 (100.0) |  | 2 (28.6) | 5 (71.4) |  | 4 (80.0) | 1 (20.0) |  | 0 (0) | | 0 (0) |  |
| Myoepithelial | 0 (0) | 2 (100.0) |  | 1 (50.0) | 1 (50.0) |  | 1 (100.0) | 0 (0) |  | 0 (0) | | 0 (0) |  |
| Ex pleomorphic adenoma | 0 (0) | 2 (100.0) |  | 0 (0) | 2 (100.0) |  | 2 (100.0) | 0 (0) |  | 0 (0) | | 0 (0) |  |
| Squamous cell | 2 (15.4) | 11 (84.6) |  | 6 (46.2) | 7 (53.8) |  | 4 (57.1) | 3 (42.9) |  | 2 (100.0) | | 0 (0) |  |
| Oncocytic | 0 (0) | 1 (100.0) | **0.043** | 0 (0) | 1 (100.0) | **0.014** | 1 (100.0) | 0 (0) | 0.108 | 0 (0) | | 0 (0) |  |
| Histology |  |  |  |  |  |  |  |  |  |  | |  |  |
| High-risk | 13 (16.9) | 64 (83.1) |  | 31 (40.3) | 46 (59.7) |  | 34 (73.9) | 12 (26.1) |  | 4 (80.0) | | 1 (20.0) |  |
| Low-risk | 16 (36.4) | 28 (63.6) | **0.016** | 34 (77.3) | 10 (22.7) | **<0.001** | 9 (90.0) | 1 (10.0) | 0.275 | 1 (100.0) | | 0 (0) |  |

^a^ p-values according to Pearson chi-squared test were calculated upon exclusion of missing values; ^b^ Number of events too low for statistical analysis; ^c^ Age: median 61 years, range 9-93 years.

**Supplementary table 3.** Association of tumor site and clinicopathological characteristics.

| Clinicopathological characteristics | Tumor site | | p^a^ |
| --- | --- | --- | --- |
|  | **Parotid gland** | **Other** |  |
| Total n = 127 (%) | 92 (72.4) | 35 (27.6) |  |
| Gender |  |  |  |
| Male | 54 (78.3) | 15 (21.7) |  |
| Female | 38 (65.5) | 20 (34.5) | 0.109 |
| Age^b^ |  |  |  |
| < Median | 38 (61.3) | 24 (38.7) |  |
| ≥ Median | 54 (83.1) | 11 (16.9) | **0.006** |
| T-stage |  |  |  |
| T1 | 32 (72.7) | 12 (27.3) |  |
| T2 | 13 (76.5) | 4 (23.5) |  |
| T3 | 22 (71.0) | 9 (29.0) |  |
| T4 | 19 (76.0) | 6 (24.0) | 0.965 |
| Missing values | 6 | 4 |  |
| N-stage |  |  |  |
| N0 | 55 (65.5) | 29 (34.5) |  |
| N1 | 14 (77.8) | 4 (22.2) |  |
| N2 | 22 (91.7) | 2 (8.3) |  |
| N3 | 1 (100.0) | 0 (0) | 0.068 |
| Resection margins |  |  |  |
| R0 | 50 (64.9) | 27 (35.1) |  |
| R1 | 30 (81.1) | 7 (18.9) |  |
| R2 | 6 (85.7) | 1 (14.3) | 0.139 |
| No surgery | 6 | 0 |  |
| Carcinoma histology |  |  |  |
| Acinic cell | 12 (100.0) | 0 (0) |  |
| Mucoepidermoid G1/G2 | 10 (50.0) | 10 (50.0) |  |
| Mucoepidermoid G3 | 0 (0) | 2 (100.0) |  |
| Adenoid cystic | 13 (54.2) | 11 (45.8) |  |
| Polymorphous adeno | 0 (0) | 2 (100.0) |  |
| Epithelial-myoepithelial | 2 (50.0) | 2 (50.0) |  |
| Clear cell | 1 (100.0) | 0 (0) |  |
| Basal cell adeno | 3 (75.0) | 1 (25.0) |  |
| Adeno, not otherwise specified | 24 (77.4) | 7 (22.6) |  |
| Salivary duct | 7 (100.0) | 0 (0) |  |
| Myoepithelial | 2 (100.0) | 0 (0) |  |
| Ex pleomorphic adenoma | 2 (100.0) | 0 (0) |  |
| Squamous cell | 15 (100.0) | 0 (0) |  |
| Oncocytic | 1 (100.0) | 0 (0) | **0.001** |
| Histology |  |  |  |
| High-risk | 62 (75.6) | 20 (24.4) |  |
| Low-risk | 30 (66.7) | 15 (33.3) | 0.281 |
| Surgery |  |  |  |
| Without neck dissection | 15 (51.7) | 14 (48.3) |  |
| With neck dissection | 71 (77.2) | 21 (22.8) | **0.008** |
| No primary surgery | 6 | 0 |  |
| Adjuvant radiotherapy |  |  |  |
| Postoperative radiotherapy | 32 (74.4) | 11 (25.6) |  |
| Postoperative chemoradiotherapy | 11 (84.6) | 2 (15.4) |  |
| No adjuvant treatment | 43 (66.2) | 22 (33.8) | 0.202 |
| No primary surgery | 6 | 0 |  |

^a^ p-values according to Pearson chi-squared test were calculated upon exclusion of missing values; ^b^ Age: median 61 years, range 9-93 years.

**Supplementary table 4.** Association of most frequent histological subtypes with clinicopathological characteristics.

| Clinicopathological characteristics | Acinic cell carcinoma | Mucoepidermoid carcinoma G1/G2 | Adenoid cystic carcinoma | Adenocarcinoma, not otherwise specified | Squamous cell carcinoma | p^a^ |
| --- | --- | --- | --- | --- | --- | --- |
| Total n = 102 (%) | 12 (11.8) | 20 (19.6) | 24 (23.5) | 31 (30.4) | 15 (14.7) |  |
| Gender |  |  |  |  |  |  |
| Male | 3 (5.5) | 8 (14.5) | 13 (23.6) | 22 (40.0) | 9 (16.4) |  |
| Female | 9 (19.1) | 12 (25.5) | 11 (23.4) | 9 (19.1) | 6 (12.8) | 0.051 |
| Age^b^ |  |  |  |  |  |  |
| < Median | 8 (15.4) | 16 (30.8) | 19 (36.5) | 7 (13.5) | 2 (3.8) |  |
| ≥ Median | 4 (8.0) | 4 (8.0) | 5 (10.0) | 24 (48.0) | 13 (26.0) | **<0.001** |
| T-stage |  |  |  |  |  |  |
| T1 | 8 (23.5) | 13 (38.2) | 4 (11.8) | 5 (14.7) | 4 (11.8) |  |
| T2 | 1 (7.1) | 2 (14.3) | 3 (21.4) | 3 (21.4) | 5 (35.7) |  |
| T3 | 2 (9.1) | 0 (0) | 7 (31.8) | 10 (45.5) | 3 (13.6) |  |
| T4 | 0 (0) | 2 (8.3) | 7 (29.2) | 12 (50.0) | 3 (12.5) | **<0.001** |
| Missing values | 1 | 3 | 3 | 1 | 0 |  |
| N-stage |  |  |  |  |  |  |
| N0 | 11 (16.7) | 20 (30.3) | 17 (25.8) | 11 (16.7) | 7 (10.1) |  |
| N1 | 1 (6.3) | 0 (0) | 5 (31.3) | 7 (43.8) | 3 (18.8) |  |
| N2 | 0 (0) | 0 (0) | 2 (10.5) | 13 (68.4) | 4 (21.1) |  |
| N3 | 0 (0) | 0 (0) | 0 (0) | 0 (0) | 1 (100.0) | **<0.001** |
| Resection margins |  |  |  |  |  |  |
| R0 | 8 (13.6) | 18 (30.5) | 12 (20.3) | 15 (25.4) | 6 (10.2) |  |
| R1 | 3 (9.1) | 2 (6.1) | 12 (36.4) | 11 (33.3) | 5 (15.2) |  |
| R2 | 0 (0) | 0 (0) | 0 (0) | 2 (50.0) | 2 (50.0) | 0.072 |
| No surgery | 1 | 0 | 0 | 3 | 2 |  |
| Postoperative (chemo-)radiotherapy |  |  |  |  |  |  |
| Yes | 5 (9.8) | 3 (5.9) | 15 (29.4) | 19 (37.3) | 9 (17.6) |  |
| No | 7 (13.7) | 17 (33.3) | 9 (17.6) | 12 (23.5) | 6 (11.8) | **0.084** |

^a^ p-values according to Pearson chi-squared test were calculated upon exclusion of missing values; ^b^ Age: median 61 years, range 9-93 years.
